# Indirect impacts of a highway on movement behavioral states of a threatened tortoise and implications for landscape connectivity

**DOI:** 10.1038/s41598-024-51378-z

**Published:** 2024-01-06

**Authors:** Seth Harju, Scott Cambrin, Jodi Berg

**Affiliations:** 1 Heron Ecological, LLC, P.O. Box 235, Kingston, ID 83839 USA; 2 Clark County Desert Conservation Program, 4701 W Russell Rd, Las Vegas, NV 89118 USA; 3 Alta Science and Engineering, 220 E Fifth St, Suite 325, Moscow, ID 83843 USA

**Keywords:** Ecology, Conservation biology

## Abstract

Roads have often been identified as barriers to the movement of free-ranging animals. However, whether restoration of landscape connectivity across roadways can mitigate barriers to movement is insufficiently understood in light of indirect effects of roads on wildlife movement. We GPS-tagged free-ranging Mojave desert tortoises (*Gopherus agassizii*) to quantify movement behavioral states using hidden Markov models in relation to a major highway and to document use of existing, permeable culverts. We then used the observed movement behaviors to parameterize simulations of tortoise movement to evaluate alternative culvert designs and placements for enhancing connectivity across the roadway. Tortoises were most active during mid-day, in warm temperatures, and when close to the highway. The highway affected transition probabilities between movement states, as females were more likely than males to switch to an energy-demanding traveling movement state, remain in that state, and move farther than usual within that state. In contrast, males were more likely than females to continue in the low-energy resting state when close to the highway, but if traveling, to travel farther than usual. We observed two highway crossings by a tagged tortoise, which was a higher rate of crossing than in simulated tortoises. Simulated crossing rates increased with culvert size and culvert density, and size and density appeared more important for crossing than if culverts were placed singly or in pairs. Existing culvert densities across the region appeared potentially sufficient for long-term genetic connectivity, but only if retrofitted to allow for tortoise access and passing. We concluded that existing highway traffic may indirectly depress tortoise populations adjacent to the highway, particularly via negative impacts to female movements, and that existing culverts in washes should be retrofitted to allow for periodic tortoise crossings to improve structural connectivity for occasional passage.

## Introduction

Humans develop and change landscapes in many ways^1^. Understanding how these changes impact animal movement is important because of the opportunities and costs that movement affords individual animals^2,3^. Movement can facilitate include relocating to better foraging or reproductive habitat, reducing exposure to predators or parasites, or maintaining genetic diversity^4–6^. Movement also includes costs because it involves exposure to a wider variety of predation and mortality risks and because it requires energy expenditure. For example, individual mule deer (*Odocoileus hemionus*) movement in response to human landscape development can increase energy expenditure up to 70%, which may result in significant reductions in population size^7,8^).

The building and use of roads are one type of human landscape development that pervasively affects animal movement. U The wide variety of both positive and negative, and both direct and indirect, impacts of roads on wildlife species’ movement can be complicated and difficult to tease apart. Positive effects on individual species include movement facilitation (e.g., wolves [*Canis lupus*] preferentially move along industrial seismic line roads during snow-free seasons, increasing access to prey^9^) or by serving as refuge from predation (e.g., moose [*Alces alces*] prefer to birth near paved roads in areas where the primary predator of neonates, brown bears [*Ursus arctos*], avoid paved roads^10^). However, negative effects of roads are much more common. Direct vehicle collisions are well-known and are responsible for hundreds of millions of vertebrate mortalities per year on roads in the U.S. alone^11,12^. Roads effectively prevent or reduce movement by acting as an impermeable or semipermeable barrier, ultimately fragmenting populations and hampering an individual’s ability to maximize fitness^3,13,14^. For example, elk (*Cervus elaphus*) simultaneously alter habitat use and move away from roads when traffic increases, resulting in the effective loss of preferred habitats^15^. Similarly, bat foraging movements are reduced nearer to major highways, effectively resulting in reduced species diversity within 1.6 km of highways^16^.

The negative impacts of roads are especially pronounced for low-density species with low reproductive rates^2^. The Mojave desert tortoise (*Gopherus agassizii*) is one such species of high conservation concern. Mojave desert tortoises occur across the Mojave and Colorado deserts of the U.S. southwest but currently face several threats to their survival as a species. Habitat destruction due to development, invasive plant species, and off-road vehicle use have resulted in a loss of crucial habitat for the tortoise^17^. Fragmentation of remaining habitat can isolate populations and limit both genetic diversity and demographic rescue^18^. Direct mortality from roads, disease, and increasing populations of human-subsidized predators have also contributed to the overall decline in the species across its range^19–21^. In addition, climate change may further exacerbate the challenges facing the Mojave desert tortoise by altering the distribution and abundance of vegetation and increasing the frequency and severity of droughts^22,23^. Due to these threats, the Mojave desert tortoise is currently listed as a threatened species under the U.S. Endangered Species Act and conservation efforts are ongoing to protect remaining habitat, monitor populations, and minimize other threats to the species as it continues to decline^24–26^.

Roads in particular can have a significant impact on Mojave desert tortoises, both via direct mortality and by creating barriers that limit movement. Roads reduce connectivity and gene flow among tortoise populations, which can reduce genetic diversity and increase the risk of local extinctions^27^. The effects of roads have led to an identified “road effect zone” where tortoise populations are decreased or altogether extirpated adjacent to roads^20,28–31^. As a response to direct mortality, current best management practices call for exclusionary fencing along highways to prevent tortoise ingress onto the highway, which has successfully reduced direct mortality^29,32^. However, large-scale exclusionary fencing has the unintended consequence of cementing the population-fragmenting nature of roads, completely curtailing long-term gene flow, population connectivity (e.g., dispersal), and adaptive range shifts in response to climate change^33^. In addition to being an impermeable barrier, there is conflicting evidence on how roads indirectly affect movement of Mojave desert tortoises. In some cases, tortoises have longer steplengths when near low-traffic roads and shorter steps near highways^32,34^, but sometimes tortoises avoid roads altogether^35^. These long-term indirect impacts of exclusionary fencing on near-highway tortoise movement behaviors are not yet well-understood.

A partial resolution to the unintended consequences of road fencing is existing culverts under roadways for water drainage. Currently, within the range of Mojave desert tortoises, culverts are often built (as part of road replacements and repairs) or retrofitted (for existing culverts) with the hope that they facilitate tortoise movement under fenced highways, thus restoring connectivity^36,37^. Tortoises have been documented using culverts, but such documentation is rare and the frequency of tortoises using culverts for crossing is poorly understood. Importantly, what is currently missing is a mechanistic link between fenced roadways, the landscape connectivity that culverts may provide, and culvert functionality given altered tortoise movement behaviors adjacent to roadways.

To evaluate movement of Mojave desert tortoises in relation to a fenced highway and underlying culverts, we conducted a GPS movement study of free-ranging tortoises along U.S. Highway 95 in northern Clark County, Nevada, USA. We then used movement metrics from the analysis of free-ranging tortoises to conduct simulations on tortoise movement within the landscape, but differing the configuration of highway crossings to both assess the potential benefits of increasing connectivity and to serve as a null model on crossing rates in this landscape. Our objectives included using GPS data from the tagged tortoises to: (1) delineate and quantify behavioral movement states near the highway, (2) evaluate whether movements within and transitions between movement states were affected by proximity to the highway, (3) quantify culvert crossing rates, and (4) parameterize simulations of tortoise movements to determine expected highway crossing rates at random given the existing and potential alternative arrangements of culverts. The ultimate goal was improved understanding of the impacts of highways on tortoise movement and landscape connectivity.

## Methods

### Study area

**Figure 1:**
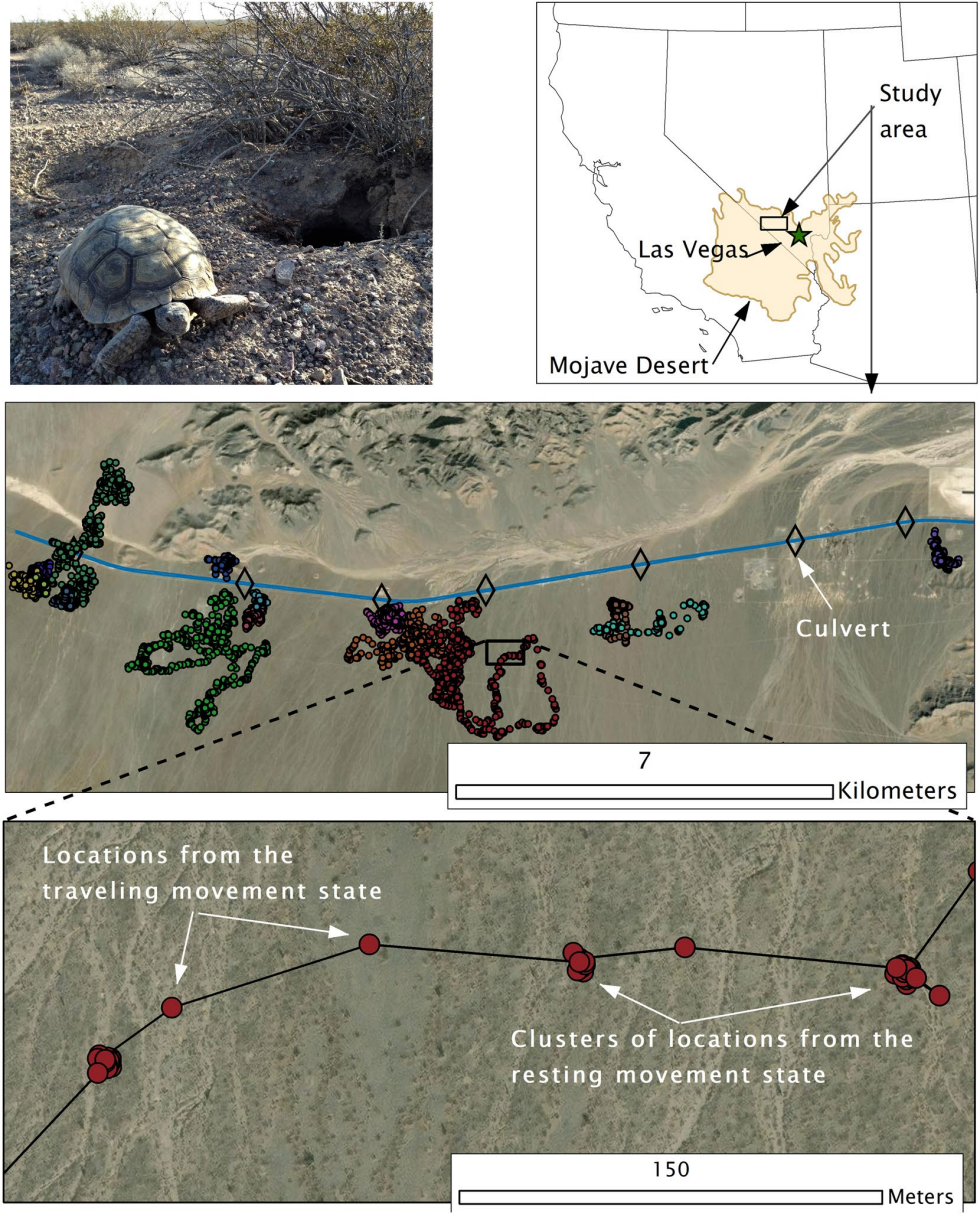
A Mojave desert tortoise, study area, GPS locations, and apparent movement states from raw data for Mojave desert tortoises along U.S. Highway 95, Clark County, NV, U.S., 2021. = Photo credit S. Cambrin.

The study area was along U.S. Highway 95 in southern Nevada, U.S. in the eastern Mojave Desert (Fig. 1). Low elevations in this portion of the Mojave Desert receive an average of 11 cm of precipitation per year in the form of winter rain and snow and summer monsoon rains^38,39^. The study area has experienced the large regional megadrought beginning in 2000 and continuing past our study period^23^. The habitat was uniform Mojave Desert scrub, which is dominated by the creosote bush (*Larrea tridentata*) and white bursage (*Ambrosia dumosa*) shrub alliance^40^. On both sides of the highway numerous shallow, dry washes were oriented from north to south.

### Field data collection

We captured, GPS-tagged, and released Mojave desert tortoises within the study area between April and August of 2021. We restricted searches and captures of tortoises to 800-m radius semicircles centered on the north and south sides of eight existing tortoise-passable culverts along the highway (i.e., we targeted tortoises with a reasonable chance of crossing the road via the nearest culvert and whose home ranges were adjacent to the highway). These searches accounted for 14 of the 15 tortoises in the study with the last tortoise initial location being ~ 300 m outside of the capture semicircles. We affixed each tortoise with a Holohil radio transmitter (RI-2B, 15.0 g, 24-month battery life) and a custom aluminum bracket that could hold one modified Mobile Action i-gotU GT-120 GPS datalogger. We modified the i-gotU units by replacing the 380 mAh factory batteries with 1000 mAh batteries. We set GPS units to record location coordinates every 30 min from date of capture until October 31st, 2021. We located tortoises by VHF radio receiver once per week during the active season (March–October) and changed the GPS dataloggers every 3–4 weeks. When tortoises died from natural causes (mostly predation) their units were not re-deployed.

In 2021, we conducted field assessments of all culverts associated with tortoise exclusionary fencing within Clark County, NV. Each culvert was assessed for its passability by a desert tortoise, as well as whether the culvert was attached to the exclusionary fencing. These data were recorded for each side of the culvert independently and then later combined using ArcGIS 10.8.1.

### Statistical analysis

#### Movement states

We used Hidden Markov Models (HMMs) to assign the GPS locations of tortoises to one of a set of mutually exclusive latent behavioral movement states based on two movement metrics: step length and turning angle. We calculated step length as the straight-line distance (m) between consecutive 30-min GPS locations and turning angle as the difference in the angle (radians) of two consecutive straight-line steps. We implemented HMMs using the ‘momentuHMM’ package^41^ in Program R v 4.2^42^ and used the prepData function to calculate step length and turning angle. We assumed that step lengths followed a gamma distribution and that turning angles followed a von Mises distribution. The gamma distribution is a flexible strictly positive distribution (i.e., negative step lengths are not possible) and the von Mises distribution is a common distribution for circular data, such as turning angles^43^. We assumed that observed locations came from one of *k* mutually exclusive user-defined latent states and that the sequence of states is generated by a Markov chain. This means that the state from which a location is sampled at time *t* is related to the state at time *t-1*, usually with a tendency to remain in state *k* for some length of time before switching to another state. The probabilities of switching between states are referred to as transition probabilities and it is often of high biological interest to model state transition probabilities as a function of environmental covariates^41,43^.

We used Akaike’s Information Criterion (AIC) to determine the optimal number of baseline latent movement states, either a single indistinguishable state or two mutually exclusive states. We set a maximum of two movement states because examination of the sequential GPS locations strongly indicated the existence of two states (Fig. 1) and because more than two states are difficult to distinguish using HMMs with only two movement metrics^44^. We characterized a two-state model as being composed of a resting state encompassing any localized behaviors, including resting, feeding, basking, burrowing, and aestivating (e.g., during summer), and a traveling state encompassing all non-localized moving behaviors, such as foraging, dispersal, mate-seeking, etc.

We fit the HMM models in three steps. First, we evaluated seven biological models on step length and the standard deviation (SD) in step length, testing all possible combinations of ambient temperature, individual sex, and cosine (hour of the day), using AIC to select the most parsimonious model. Second, we evaluated the same seven biological models on transition probabilities between movement states, and then combined the best models for steps and transition probabilities into a final ‘best’ biological movement model for within-state movements and transition probabilities between states. Third, we evaluated the utility of including a log (distance to highway) predictor on step length and SD, transition probabilities, both steps and transition probabilities, and finally, on whether there was an interaction effect between individual sex and the effect of proximity to the highway on both steps and transition probabilities. Competing models were evaluated using AIC and relative weights of evidence in favor of the models that were tested^45^. Ad-hoc analyses testing for seasonal variation (i.e., a quadratic trend as a function of Julian day) indicated no relationships between movement metrics or transition rates (e.g., aestivation in peak summer) and Julian day within our data set and thus we did not further consider seasonal patterns.

#### Crossing rates

We assessed highway crossing in two ways. First, we counted the number of observed crossings of GPS-tagged tortoises. Second, we used movement characteristics of GPS-tagged tortoises to simulate movement paths of hypothetical desert tortoises in relation to an impermeable highway with intermittent permeable corridors (i.e., culverts). To reflect heterogeneous landscape resistance to movement for desert tortoises, we included a modified model of landscape connectivity for desert tortoises^46,47^. We re-scaled the connectivity layer from 0 (high connectivity, or low resistance) to 1 (no connectivity, or high resistance/impermeable) and merged the layer with layers of rasterized roads (classified as 1) and rasterized culverts (classification ranged from 0.05 inside the culvert to 0.25 just outside the culvert to reflect potential ‘attractiveness’ of the culvert as a burrow surrogate or as a way past the fenced highway). We set the raster grid resolution at 0.6 m, the width of the smallest culvert size in the study area.

We simulated desert tortoise movements using parameter estimates from the simple two-state HMM described above. Each movement state had its own set of movement parameters. For the resting state, we used a true random walk model, with a step length of 1 raster grid cell (i.e., 0.6 m). This was to encompass short, localized movements in addition to true stationarity as well as GPS error associated with true stationarity. For the traveling state, we specified a correlated random walk with maximum step lengths of 45 m (to avoid simulated tortoises ‘leaping’ over the highway barrier) and an observed correlation of turning angles between successive steps of 0.707. The probability of transitioning from the resting to the traveling state was 0.098 at each time step, and conversely from the traveling to the resting state was 0.228 (both values extracted from the HMM, thus reflecting real movement parameters in the local tortoise population). We defined the perceptual range of the simulated tortoises (i.e., the range via which they could perceive the underlying resistance surface and thus make shorter step lengths or avoid high-resistance raster cells) as a Gaussian kernel centered on the current location with a standard deviation of 8 m (e.g., 66% of the perceived resistance cells were within ~ 25 map units). This reflected declining perception of limitations to movement as distance increased and is based on observed perception distances in other large tortoise species^48,49^.

Initial abundance of simulated tortoises was determined based on unpublished mark-recapture abundance analyses at these plots. We generated random numbers of starting abundance at each plot, drawing from a negative binomial distribution parameterized as mean = 70 and size = 13.08. To reflect the movements and potential encounters with culverts that free-ranging desert tortoises experience, the starting locations for simulated tortoises were randomly generated within the plots. For each simulation, the tortoises were set to step at 30-min intervals for 730 days (i.e., 2 years), ignoring the fact that desert tortoises spend the majority of the year aestivating or brumating underground (i.e., the ecological equivalent of simulated movements was greater than 2 years).

We evaluated results based on the number of times a simulated tortoise crossed the road under three densities of culverts, two culvert widths, and culverts placed singly or doubly (i.e. in pairs). Culvert densities ranged from the actual density existing on the landscape (7 single culverts = 0.40 culverts/km or 2.5 km between culvert locations), to a medium density (7 paired culverts = 0.80 culverts/km, and 14 single culverts or paired = 0.80 and 1.60 culverts/km, respectively, or 1.25 km between culvert locations) and a higher density of 28 single culverts (1.60 culverts/km = 625 m between culverts). Culvert widths included 61 cm (the smallest culvert size in the study area) and a wider culvert of 183 cm (the most common width of box culverts used in the study area). The combinations of culvert density, width, and singular or double placement meant tortoises could cross under ten different scenarios. We repeated simulations 1,000 times for each of the scenarios and conducted simulations within package ‘SiMRiv’ in Program R (v 4.0), which allows for multi-state movement simulations in heterogeneous environments with barriers to movement^50^.

## Results

**Figure 2:**
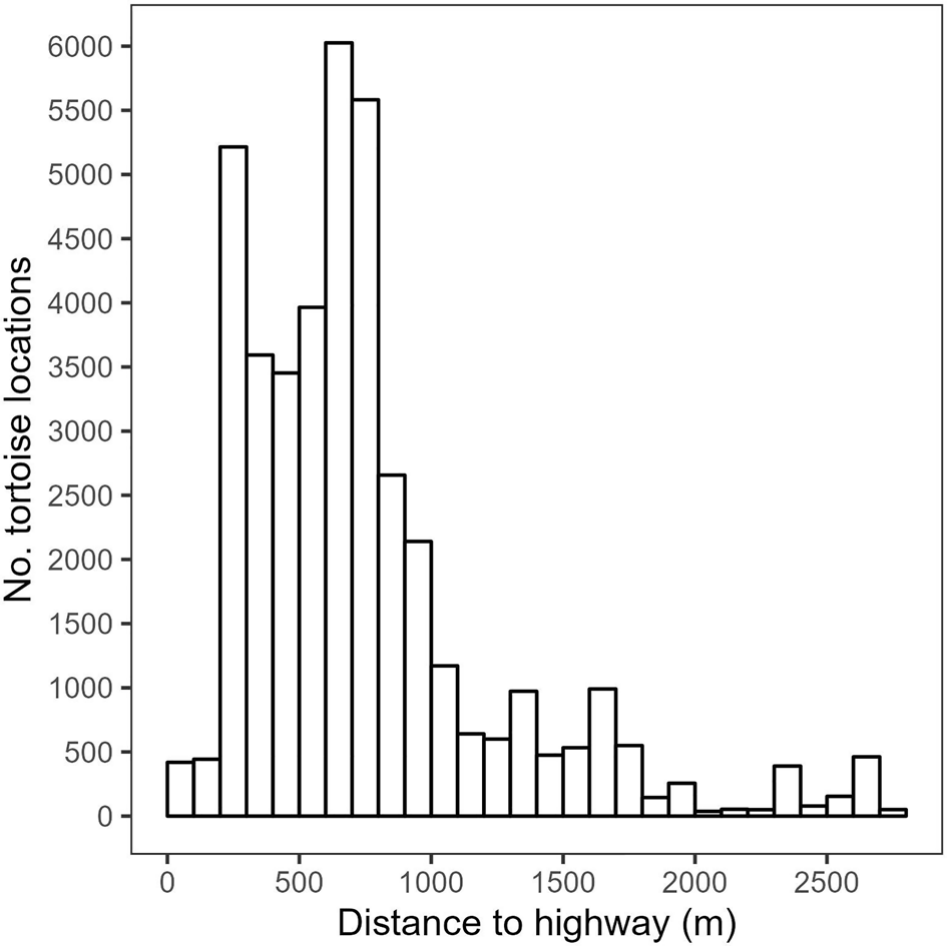
Histogram of distances from each Mojave desert tortoise’s GPS location to U.S. Highway 95 in northwestern Clark County, NV, April–October 9, 2021.

Fifteen adult tortoises (nine males and six females) were captured and outfitted with GPS units. Fourteen tortoises were captured between 4/3/21 and 5/14/21; the fifteenth tortoise was captured on 8/19/21. There was a total of 1298 tortoise-days with recorded GPS locations. Two tortoises died in May 2021, one tortoise died in June, four in July, one in August, and two in October. Two tortoises went missing (one in July 2021 and one in September 2021). Three female tortoises were alive at the end of our monitoring period. Tortoise locations were generally between 200 and 1000 m from the highway (Fig. 2).

There were approximately 340 km of paved roads in Clark County with tortoise exclusionary fencing. There were also 720 existing culverts. Of the existing culverts, only 89 (12.4%) were tied in with the fencing and thus available for use by tortoises. Attached culverts were far apart, with a mean distance apart of 3.86 km (SD 4.72 km). For all culverts, the mean distance apart was 472 m (SD 577 m).

### Movement states

Initial assessment of a baseline biological movement model found overwhelming support for a model with two underlying behavioral states versus a single state (∆AIC = 33,413.8). Within a two-state model, intrastate step lengths and SD in step lengths were best explained by a model comprised of cosine (hour of day), ambient temperature, and sex of the tortoise as predictor variables (∆AIC ≥ 41.9, Table 1). Transition probabilities between movement states were also best explained by a model with cosine (hour of day), ambient temperature, and sex of the tortoise as predictor variables (∆AIC ≥ 30.1, Table 1). Combined, the best overall model was therefore one where hour of day, temperature, and sex influenced both movement behaviors within states and transition probabilities between states.

**Table 1:** Model selection results for baseline biological models on movement within, and transition probabilities between, two latent movement states for Mojave desert tortoises in northwestern Clark County, NV, U.S., 2021.

Model component	Model	AIC^a^	ΔAIC^b^
Step length and SD of step length	Hour + Temp + Sex	375,440.9	0
	Hour + Sex	375,482.8	41.9
	Hour + Temp	375,842.2	401.3
	Hour	375,914.8	473.9
	Temp + Sex	376,334.2	893.3
	Sex	376,645.3	1204.4
	Temp	376,701.1	1260.2
Transition probabilities	Hour + Temp + Sex	376,306.4	0.0
	Hour + Sex	376,336.5	30.1
	Hour + Temp	376,454.2	147.8
	Hour	376,481.0	174.6
	Temp + Sex	376,821.7	515.3
	Sex	376,925.0	618.6
	Temp	376,958.9	652.5

^a^Akaike’s Information Criterion.

^b^Difference in AIC for a given model from the model with the lowest AIC value.

Every model where log(distance to highway) was included as any predictor in addition to the best biological model performed better than the best biological model performed alone (∆AIC ≥ 12.1, Table 2). Of the biological + highway models, the best performing model (∆AIC = 22.6) was one where there was an interaction of individual sex on the distance to highway effect, affecting movement behaviors both within states and transition probabilities between states and did so differently for males and females (Table 2).

**Table 2:** Model selection results for additional highway and sex effects on movement within, and transition probabilities between, two latent movement states for Mojave desert tortoises in northwestern Clark County, NV, U.S., 2021.

Model	AIC^a^	ΔAIC^b^
Step (Biol^c^ + Hwy*Sex) + Transition (Biol + Hwy*Sex)	375,080.4	0
Step (Biol + Hwy) + Transition (Biol + Hwy)	375,103.0	22.6
Step (Biol + Hwy) + Transition (Biol)	375,126.1	45.7
Step (Biol) + Transition (Biol + Hwy)	375,133.7	53.3
Step (Biol) + Transition (Biol)	375,145.8	65.4

^a^Akaike’s Information Criterion.

^b^Difference in AIC for a given model from the model with the lowest AIC value.

^c^’Best’ baseline biological movement model.

**Figure 3:**
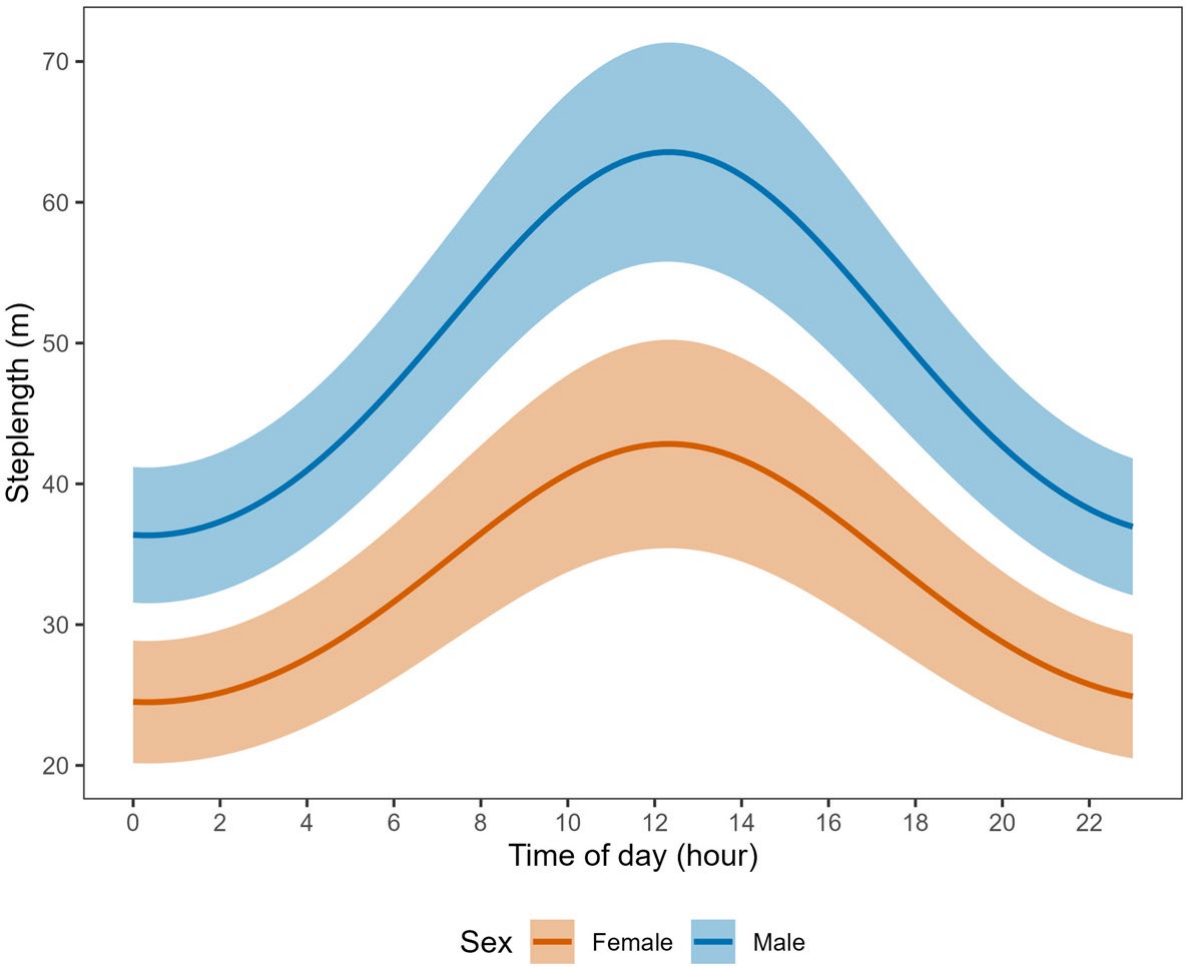
Mean step length (per 30-min GPS location interval) for Mojave desert tortoises during the traveling movement state over the 24-h diel period, Clark County, NV, April–October, 2021.

**Figure 4:**
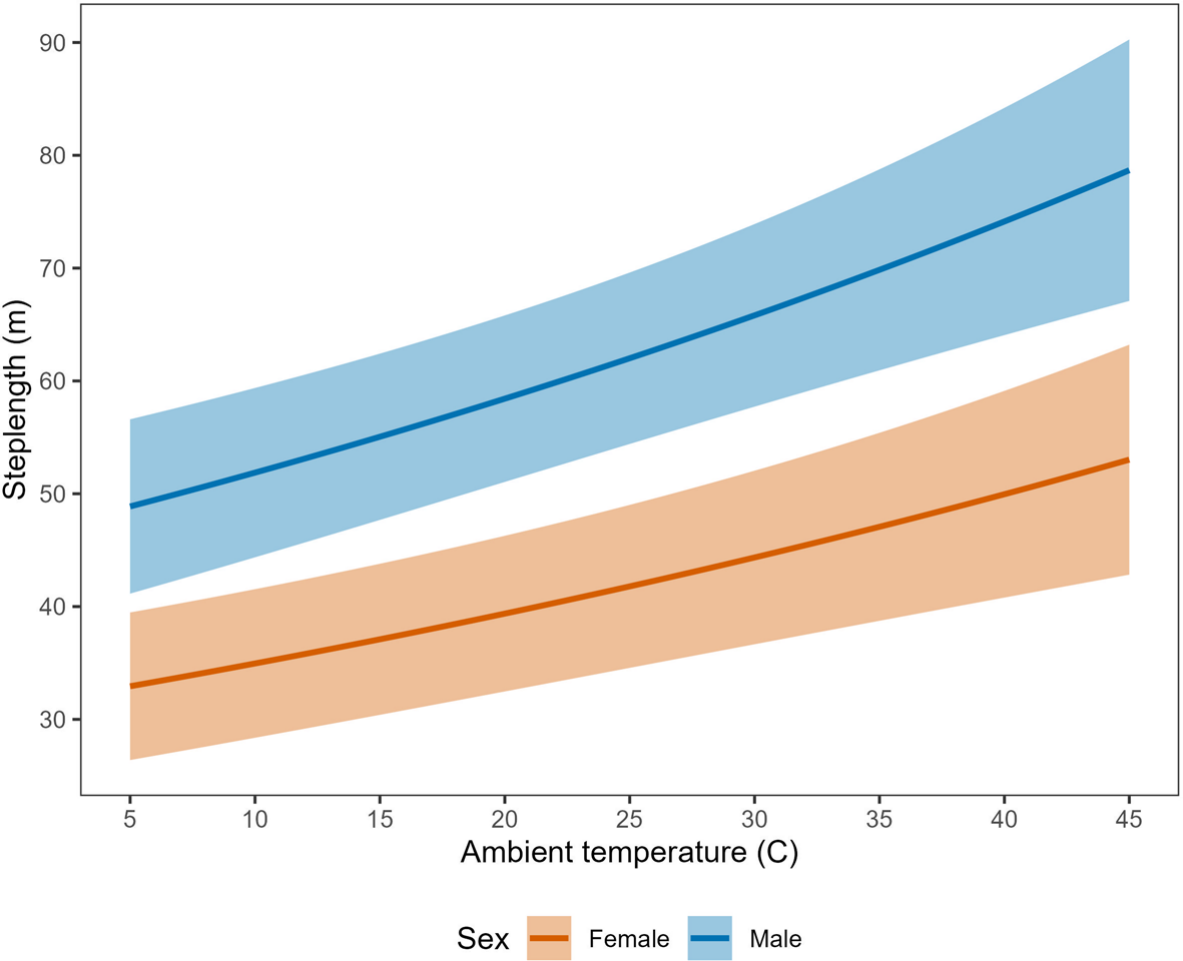
Mean step length (per 30-min GPS location interval) for Mojave desert tortoises during the traveling movement state in relation to ambient temperature, Clark County, NV, U.S., April–October 2021.

**Figure 5:**
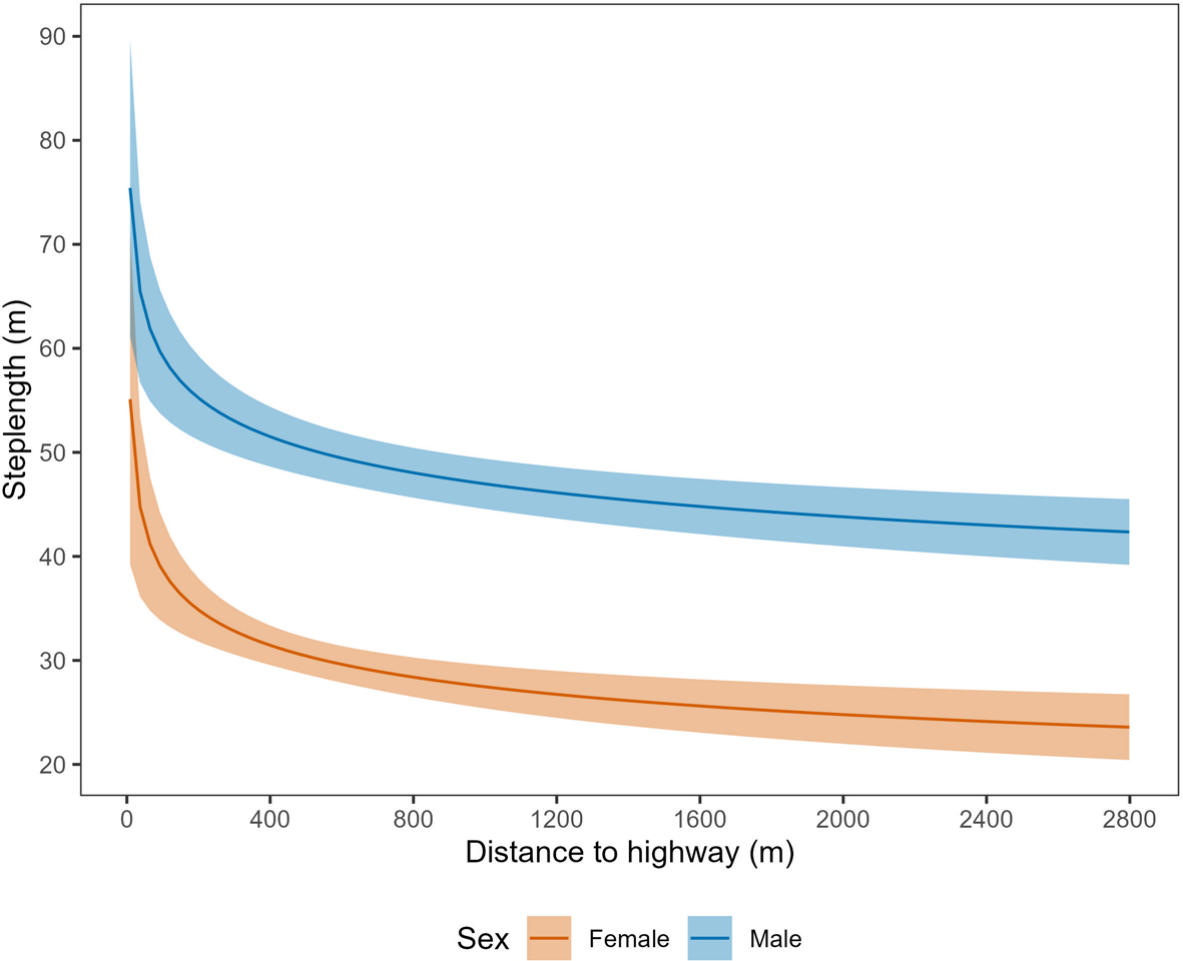
Mean step length (per 30-min GPS location interval) for Mojave desert tortoises during the traveling movement state in relation to proximity to U.S. Highway 95, Clark County, NV, U.S., April–October 2021.

**Figure 6:**
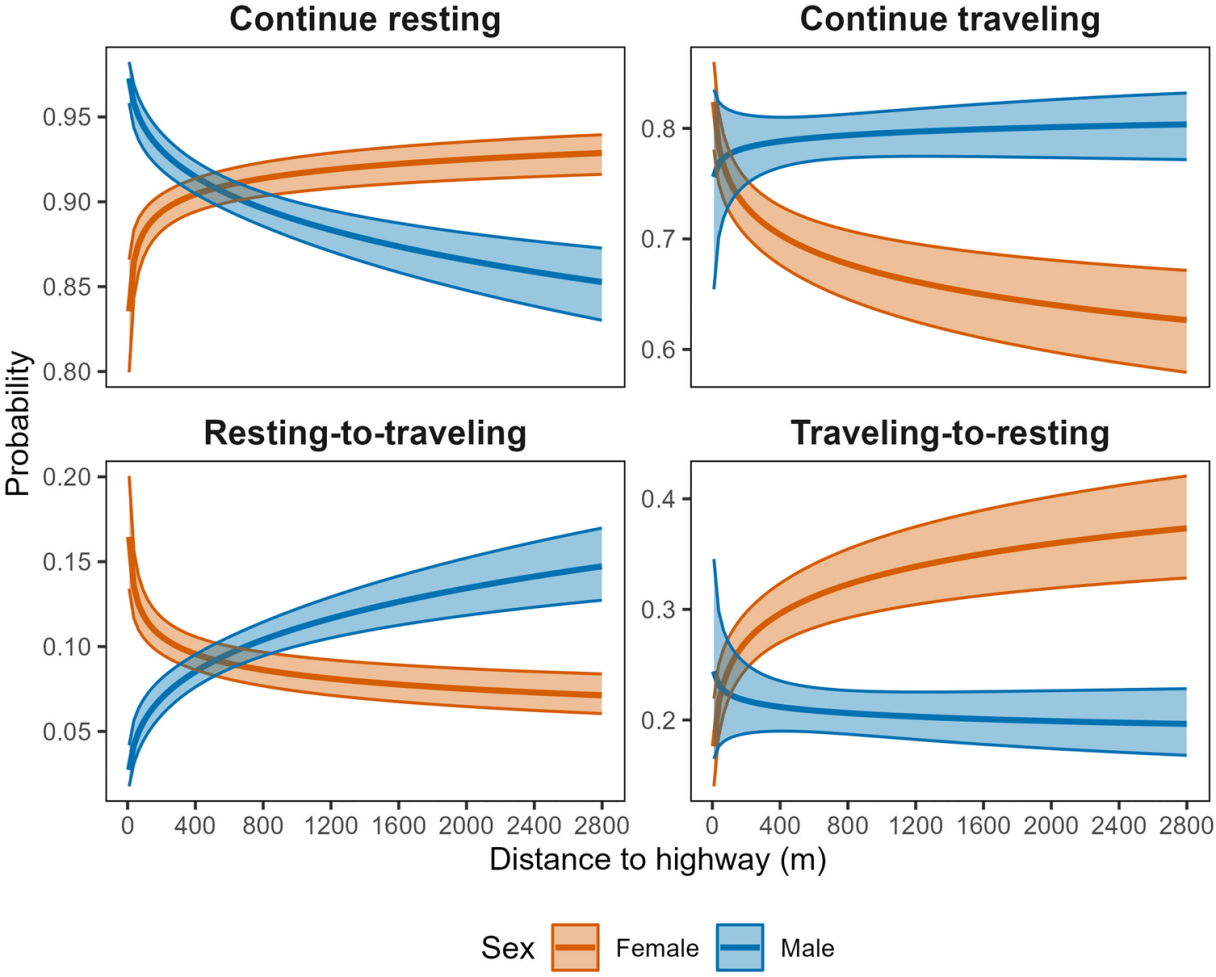
Probability of remaining within or switching between two mutually exclusive movement states for Mojave desert tortoises in relation to proximity to U.S. Highway 95, Clark County, NV, U.S., April–October 2021.

Average step lengths at mean covariate values in the resting state were equivalent between male (4.4 m, 95% CI 4.3–4.5 m) and female (4.2 m, 95% CI 4.1–4.4 m) tortoises. In the traveling state, however, males moved notably farther, with a mean 30-min distance of 47.8 m (95% CI 45.5–50.1 m) compared to 28.4 m (95% CI 26.6–30.1 m) for female tortoises. Desert tortoises exhibited diel variation in step length, moving longer distances per 30-min location interval during the middle of the day (e.g., 1000–1500 h; Fig. 3). Step length was also a function of ambient temperature, increasing by ~ 50% from 5 to 45 °C (our minimum and maximum observed temperatures; Fig. 4). The top model indicated that male and female tortoises responded differently to proximity to the highway, with males showing slightly weaker decline in step lengths at greater distances than females (difference in avoidance β = 0.047, 95% CI − 0.020–0.115; Fig. 5). The pronounced divergent effect of proximity to the highway on male and female tortoises was on transition probabilities. When in the resting state, females near the highway were less likely to stay in the resting state and more likely to switch to the traveling state (Fig. 6). When resting, males near the highway were more likely to continue resting and less likely to switch to the traveling state. Conversely, when in the traveling state, females near the highway were more likely to continue traveling and less likely to switch down into the resting state. When in the traveling state, males were equally likely to continue traveling or switch to resting regardless of proximity to the highway (Fig. 6).

### Crossing rates

The pattern of simulated tortoise movements matched the pattern of free-ranging tortoises, alternating between highly clustered GPS locations representing the resting state and longer step lengths representing the traveling state. For the monitored free-ranging tortoises, only two crossing events (0.005% of steps) occurred: a single male crossed from the north side of a culvert to the south side of Highway 95 on April 14, 2021, and returned through the same culvert back to the north side of the highway on June 30th, 2021.

Simulated tortoise use of culverts was even rarer than that of free-ranging tortoises, despite specifically enhancing the apparent attractiveness of the culverts to the simulated tortoises (e.g., lower resistance than that of the surrounding landscape). If simulated tortoises crossed the highway at the same rate as free-ranging tortoises, we would have expected ~ 123 crossings per simulation (i.e., an average of 2,451,644 simulated steps * 0.005% of steps resulting in an observed crossing/100). Instead, simulated tortoises crossed at varying rates, depending on the combination of width, number, and placement of culverts (Table 3). As expected, average crossing rates across tortoises and simulations increased with number of culverts and culvert width, with the highest crossing rates occurring with 28 72″ culverts, placed either singly (0.00015%, range: 0–0.00057%) or in 14 pairs (0.00016%, range: 0–0.00063%). Average crossing rates were similar between single culverts and those placed in pairs (e.g., crossing rates for 14 single culverts and 7 paired culverts = 0.00003%, and crossing rates for 28 single culverts and 14 paired culverts = 0.00005%).

**Table 3:** Mean crossing rates (%, with minimum–maximum) of simulated Mojave desert tortoises across varying numbers of individuals and 1000 simulations for varying densities and width of culverts placed under U. S. Highway 95, Clark County, NV.

Number of culverts	Culvert width	
	0.6 m	1.8 m
7	0.00001 (0**–**0.00015)	0.00004 (0**–**0.00039)
14	0.00003 (0**–**0.00021)	0.00008 (0**–**0.00039)
28	0.00005 (0**–**0.00034)	0.00015 (0**–**0.00057)
Pairs 7 (14 total)	0.00003 (0**–**0.00027)	0.00009 (0**–**0.00052)
Pairs 14 (28 total)	0.00005 (0**–**0.00036)	0.00016 (0**–**0.00063)

## Discussion

We found that the best biological movement model for desert tortoises included hour of day, temperature, and sex as predictor variables for both intrastate step lengths and transition probabilities between states. Males traveled farther than females in the traveling state, and step length varied diurnally and with temperature. Proximity to the highway had different effects on males and females, with females being less likely to stay in the resting state and more likely to switch to and stay within the traveling state near the highway, while males were more likely to continue resting.

Proximity to the highway was associated with dramatically higher movement rates for both males and females, even after accounting for other endogenous (i.e., sex) and exogenous (i.e., ambient temperature) factors. Within this finding, differential impacts of highway proximity to female versus male tortoises likely have the highest conservation implications. When females were nearer to the highway, they were more likely to switch to and stay within the more energy demanding movement state. Further, when in this state, they moved even greater distances than when in the traveling state and far from the highway. This finding was opposite that of other recent research, which found that Mojave desert tortoises moved shorter distances when near a highway^34^. We hypothesize that the difference may be due to either (a) analysis design, as Hromada et al.^34^ pooled males and females for their distance to highway covariate and did not interact sex*transition probability*distance to highway, or (b) forage differences, as their highway site was on the western side of the tortoise’s range which is characterized by lower elevation and drier precipitation regimes than our study in the Eastern Mojave^38,40,51^.

Previous research has found that Mojave desert tortoises use behavioral plasticity as a way to adapt to variable resource pulses (e.g., ephemeral food plants) and costs (e.g., drought). For example, tortoises spend more time belowground and move considerably less when aboveground during drought years to minimize water loss^32,52,53^. In this study the highway is possibly “counteracting” normal behavioral regulation in response to forage, water availability, and temperature, causing female tortoises near the highway to expend considerably more energy with consequent water loss than they otherwise would (i.e., by pushing them into the energy-demanding movement state). This is likely detrimental to female tortoise survival near the highway, as increased movements drive female tortoises into water-limited conditions known to dramatically reduce survival and because increased activity aboveground increases predation risk^54–56^. This impact could also provide a partial explanation for previously noted road effect zones for the Mojave desert tortoise, whereby reduced density and lack of mature adults is not necessarily solely a function of historic direct mortality^20^.

Previous research identifying “road effect” zones for the Mojave desert tortoise predominantly concluded that direct mortality of local populations was the most likely cause of lower tortoise abundance near highways^20,28^. Roadside exclusionary fencing may alleviate this mechanism, but recent tracking of free-moving Mojave desert tortoises has continued to find avoidance of roads or altered movement near roads^32,34,35,57^. The mechanisms behind this indirect effect may be noise or vibrations permeating from the highway outwards. Noise from roads has widespread and profound negative impacts on wildlife populations across taxa^58^. Road noise itself, separate from other road impacts, has been shown to displace a third of the resident avian population, with pronounced negative body condition effects on remaining individuals^59^. Loud noises (e.g., airplanes) elicit defensive behaviors in desert Mojave desert tortoises^60^. Physical vibrations may also agitate tortoises near roads, as juvenile gopher tortoises (*Gopherus polyphemus*) use vibrations as a cue to flee potential predators^61^. Improved engineering and materials techniques are being developed to reduce vibrations and road noise, and their incorporation when highways are resurfaced could lessen this impact^62,63^. Alternatively, separate conservation measures adjacent to major roads, such as habitat restoration, shade structures, or water catchments, could ameliorate the negative indirect impacts of the road.

Our results have mixed implications for landscape connectivity. The negative impact of the highway on female movement can reduce connectivity by expanding the road effect beyond the fence, depressing local populations and thus functioning as a wider fragmentation barrier. However, we did document a tortoise crossing the highway twice via a culvert. Such short-term excursions across linear barriers have been previously documented in Mojave desert tortoises, and indicate that the road effect on tortoise movement did not render the highway as a completely impermeable barrier^34–36^.

We note that this study did have a majority of tortoise locations within the road effect zone. This is because we specifically designed this study to target animals close to the road and to ignore individuals that would not be impacted by the road or have any likelihood of crossing, and thus should not be construed as tortoises ‘selecting’ for proximity to the highway. We also note that the majority of our 15 tagged tortoises died (n = 10) or went missing (n = 2) during our 7-month study period. This is an unusually high mortality rate for Mojave desert tortoises, who typically have annual survival rates between 0.90 and 1.00^64–66^. However, mortality can be dramatically higher (e.g., up to 40% mortality) in a single year irrespective of road proximity^54,67^. Because we intentionally focused on tagging tortoises near the highway, we are unable to determine if our high observed mortality was due to proximity to the highway^20^, was due to regional drought-based mortality factors^54^, an interaction of drought and proximity to the highway, or other unobserved factors.

Simulated tortoises used culverts less frequently than free-ranging tortoises, but increasing the number and width of culverts may help increase crossings and partially mitigate the negative impact of highways on tortoise movement. Edwards et al.^68^ suggested that historic populations of the closely related Sonoran desert tortoise (*Gopherus morafkai*) maintained sufficient genetic diversity with only a single migrant per generation. Although recent genetic analyses of Mojave desert tortoises found a genetic impact of a highway^27^, perhaps the culverts can ameliorate the impact to some extent. More culvert crossings, particularly when accessible and tied into exclusionary fencing, can result in enhanced connectivity and a reduced barrier effect of the highway^35,57,69,70^.

Culverts, by design, are placed in low depressions and washes to allow water passage. Washes are a preferred habitat for tortoises as they have been shown to use them as foraging and movement corridors^46,58,71–73^. Even in the absence of fencing, Mojave desert tortoises still cross roads at the intersection with washes^35,57^. This indicates that new culverts do not need to be installed in upland desert areas around the washes as they may not add much to connectivity and that retrofitting currently in-use culverts so that they can be used for both animal connectivity and water flow would be the best course of action. The United States Fish and Wildlife Service, the U.S. agency responsible for recovery of the Mojave desert tortoise under the Endangered Species Act, recommends crossings be spaced as close to 670 m apart when possible^74^. Our simulations verified these estimates as the highest crossing rates were achieved in the scenarios with culverts ~ 625 m apart. Currently, of the 12.4% of existing culverts within Clark County, NV, that are attached to the tortoise exclusionary fencing, only 14.6% fall within the recommended spacing of 670 m apart. If all existing culverts were tied into the exclusionary fencing and thus available for tortoise use, 84.2% of culverts would meet the recommended spacing. Attaching fencing to culverts is a vital management action that should be undertaken in Southern Nevada and in other places where tortoise exclusionary fencing occurs to restore connectivity and ameliorate some of the indirect impacts of highways.

## Conclusions

Exclusionary fencing can reduce direct mortality of animals on roads, but indirect negative road impacts remain. Individual animals may still alter movement behaviors in proximity to the road, leading to negative population consequences. Nonetheless, road crossing structures can be used to restore connectivity of populations if a sufficient number of structures are available for animals to use. For Mojave desert tortoises, functional culvert density should be increased to better restore connectivity. One remaining question is whether technical improvements in road construction (e.g., reduced vibration) or alternative conservation measures adjacent to roads can ameliorate the road’s negative indirect impacts on female Mojave desert tortoises.

## Data Availability

The datasets generated and/or analysed during the current study are not publicly available due to the study species being federally listed as Threatened and of high value to illegal collection, but are available from the corresponding author on reasonable request.

## References

[1] Kerr JT, Currie DJ (1995). Effects of human activity on global extinction risk. Cons. Biol..

[2] Fahrig L, Rytwinski T (2009). Effects of roads on animal abundance: An empirical review and synthesis. Ecol. Soc..

[3] Beyer HL (2016). ‘You shall not pass!’: Quantifying barrier permeability and proximity avoidance by animals. J. Anim. Ecol..

[4] Cushman SA, Lewis JS (2010). Movement behavior explains genetic differentiation in American black bears. Landsc. Ecol..

[5] Fryxell JM, Sinclair ARE (1998). Causes and consequences of migration by large herbivores. Trends Ecol. Evol..

[6] Mysterud A (2011). Partial migration in expanding red deer populations at northern latitudes—a role for density dependence?. Oikos.

[7] Sawyer H, Korfanta NM, Nielson RM, Monteith KL, Strickland D (2017). Mule deer and energy development—long-term trends of habituation and abundance. Glob. Chang. Biol..

[8] Chambers S (2022). Conflict of energies: Spatially modeling mule deer caloric expenditure in response to oil and gas development. Landsc. Ecol..

[9] Latham ADM, Latham MC, Boyce MS, Boutin S (2011). Movement responses by wolves to industrial linear features and their effect on woodland caribou in northeastern Alberta. Ecol. Appl..

[10] Berger J (2007). Fear, human shields and the redistribution of prey and predators in protected areas. Biol. Lett..

[11] Loss SR, Will T, Marra PP (2015). Direct mortality of birds from anthropogenic causes. Annu. Rev. Ecol. Evol. Syst..

[12] Shilling F (2021). A reprieve from US wildlife mortality on roads during the COVID-19 pandemic. Biol. Conserv..

[13] Seidler RG, Long RA, Berger J, Bergen S, Beckmann JP (2015). Identifying impediments to long-distance mammal migrations. Cons. Biol..

[14] Bennett VJ (2017). Effects of road density and pattern on the conservation of species and biodiversity. Curr. Landsc. Ecol. Rep..

[15] Buchanan CB, Beck JL, Bills TE, Miller SN (2014). Seasonal resource selection and distributional response by elk to development of a natural gas field. Rangel. Ecol. Manag..

[16] Berthinussen A, Altringham J (2012). The effect of a major road on bat activity and diversity. J. Appl. Ecol..

[17] Darst CR (2013). A strategy for prioritizing threats and recovery actions for at-risk species. Environ. Manage..

[18] Lecomte J, Boudjemadi K, Sarrazin F, Cally K, Clobert J (2004). Connectivity and homogenisation of population sizes: An experimental approach in *Lacerta vivipara*. J. Anim. Ecol..

[19] Kristan WB, Boarman WI (2003). Spatial pattern of risk of common raven predation on desert tortoises. Ecology.

[20] Nafus MG, Tuberville TD, Buhlmann KA, Todd BD (2013). Relative abundance and demographic structure of Agassiz’s desert tortoise (*Gopherus agassizii*) along roads of varying size and traffic volume. Biol. Conserv..

[21] Harju SM (2021). Estimating trends of common raven populations in North America, 1966–2018. Hum-Wildl. Interact..

[22] Hughes MK, Diaz HF (2008). Climate variability and change in the drylands of Western North America. Glob. Planet. Change.

[23] Williams AP, Cook BI, Smerdon JE (2022). Rapid intensification of the emerging southwestern North American megadrought in 2020–2021. Nat. Clim. Change..

[24] United States Fish and Wildlife Service (USFWS) (1990). Determination of threatened status for the Mojave population of the desert tortoise. U.S Federal Regist.

[25] Allison LJ, McLuckie AM (2018). Population trends in Mojave desert tortoises (*Gopherus agassizii*). Herpetol. Conserv. Bio..

[26] Kissel AM (2023). Range-wide occupancy trends for the Mojave desert tortoise (*Gopherus agassizii*). Ecosphere.

[27] Dutcher KE (2020). Genes in space: What Mojave desert tortoise genetics can tell us about landscape connectivity. Conser. Genet..

[28] Boarman WI, Sazaki M (2006). A highway's road-effect zone for desert tortoises (*Gopherus agassizii*). J. Arid Environ..

[29] Peaden JM, Tuberville TD, Buhlmann KA, Nafus MG, Todd BD (2015). Delimiting road-effect zones for threatened species: Implications for mitigation fencing. Wildl. Res..

[30] Paterson JE (2019). Road avoidance and its energetic consequences for reptiles. Ecol. Evol..

[31] Averill-Murray RC, Allison LJ (2023). Travel management planning for wildlife with a case study on the Mojave desert tortoise. J. Fish Wildl. Manag..

[32] Sadoti G, Gray ME, Farnsworth ML, Dickson BG (2017). Discriminating patterns and drivers of multiscale movement in herpetofauna: The dynamic and changing environment of the Mojave desert tortoise. Ecol. Evol..

[33] Barrows CW, Henen BT, Karl AE (2016). Identifying climate refugia: A framework to inform conservation strategies for Agassiz’s desert tortoise in a warmer future. Chelonian Conserv. Biol..

[34] Hromada SJ (2023). Linear and landscape disturbances alter Mojave desert tortoise movement behavior. Front. Ecol. Evol..

[35] Hromada SJ (2020). Using movement to inform conservation corridor design for Mojave desert tortoise. Mov. Ecol..

[36] Boarman WI, Beigel ML, Goodlett GC, Sazaki M (1998). A passive integrated transponder system for tracking animal movements. Wildl. Soc. Bull..

[37] Jarvis LE, Hartup M, Petrovan SO (2019). Road mitigation using tunnels and fences promotes site connectivity and population expansion for a protected amphibian. Eur. J. Wildl. Res..

[38] Germano DJ, Bury RB, Esque TC, Fritts TH, Medica PA, Bury RB, Germano DJ (1994). Range and habitats of the desert tortoise. Biology of North American Tortoises.

[39] Abella SR, Spencer JE, Hoines J, Nazarchyk C (2009). Assessing an exotic plant surveying program in the Mojave Desert, Clark County, Nevada. USA. Environ. Monit. Assess..

[40] Keeler-Wolf, T. Mojave desert vegetation. in *Atlas of the Biodiversity of California*. 66–67 (California Department of Fish and Game, 2003).

[41] McClintock BT, Michelot T (2018). momentuHMM: R package for generalized hidden Markov models of animal movement. Methods Ecol. Evol..

[42] R Core Team. R: A language and environment for statistical computing. https://www.R-project.org/ (2022)

[43] Michelot T, Langrock R, Patterson TA (2016). moveHMM: An R package for the statistical modelling of animal movement data using hidden Markov models. Methods Ecol. Evol..

[44] McClintock BT, London JM, Cameron MR, Boveng PL (2017). Bridging the gaps in animal movement: Hidden behaviors and ecological relationships revealed by integrated data streams. Ecosphere.

[45] Burnham KP, Anderson DR (2002). Model Selection and Multimodel Inference.

[46] Gray ME, Dickson BG, Nussear KE, Esque TC, Chang T (2019). A range-wide model of contemporary, omnidirectional connectivity for the threatened Mojave desert tortoise. Ecosphere.

[47] Malcolm, J. & Lacey, L. M. Mojave desert tortoise connectivity. osf.io/qb7k6 (2019).

[48] Hayes FE, Beaman KR, Hayes WK, Harris LE (1988). Defensive behavior in the Galapagos tortoise (*Geochelone elephantopus*), with comments on the evolution of insular gigantism. Herpetologica.

[49] Walton B, Baxter RP (2019). Antipredator response of free-roaming Aldabra giant tortoise (*Aldabrachelys gigantea*) with implications for responsible wildlife tourism in the Seychelles islands. Phelsuma.

[50] Quaglietta L, Porto M (2019). SiMRiv: An R package for mechanistic simulation of individual, spatially-explicit multistate movements in rivers, heterogeneous and homogeneous spaces incorporating landscape bias. Mov. Ecol..

[51] Pietrasiak N, Drenovsky RE, Santiago LS, Graham RC (2014). Biogeomorphology of a Mojave Desert landscape—configurations and feedbacks of abiotic and biotic land surfaces during landform evolution. Geomorphology.

[52] Duda JJ, Krzysik AJ, Freilich JE (1999). Effects of drought on desert tortoise movement and activity. J. Wildl. Manage..

[53] Franks BR, Avery HW, Spotila JR (2011). Home range and movement of desert tortoises *Gopherus agassizii* in the Mojave Desert of California, USA. Endanger. Species Res..

[54] Longshore KM, Jaeger JR, Sappington JM (2003). Desert tortoise (*Gopherus agassizii*) survival at two Eastern Mojave desert sites: Death by short-term drought?. J. Herpetol..

[55] Daly JA (2019). Survival and movements of head-started Mojave desert tortoises. J. Wildl. Manage.

[56] Lovich JE (2023). High female desert tortoise mortality in the western Sonoran Desert during California’s epic 2012–2016 drought. Endanger. Species Res..

[57] Peaden JM, Nowakowski AJ, Tuberville TD, Buhlmann KA, Todd BD (2017). Effects of roads and roadside fencing on movements, space use, and carapace temperatures of a threatened tortoise. Biol. Conserv..

[58] Shannon G (2016). A synthesis of two decades of research documenting the effects of noise on wildlife. Biol. Rev..

[59] Ware HE, McClure CJW, Carlisle JD, Barber JR (2015). A phantom road experiment reveals traffic noise is an invisible source of habitat degradation. Proc. Natl. Acad. Sci..

[60] Bowles AE, Eckert SA (1997). Desert tortoises (*Gopherus agassizii*) lack an acoustic startle response: Implications for studies of noise effects. J. Acoust. Soc. Am.

[61] Radzio TA, O’Connor MP (2017). Behavior and temperature modulate a thermoregulation-predation risk trade-off in juvenile gopher tortoises. Ethology.

[62] Herman L, Withers J, Pinckney E (2006). Surface retexturing to reduce tire-road noise for existing concrete pavements. Transp. Res. Rec..

[63] Vaitkus A, Andriejauskas T, Sernas O, Cygas D, Laurinavicius A (2019). Definition of concrete and composite precast concrete pavements texture. Transport.

[64] Brand LA (2016). Mitigation-driven translocation effects on temperature, condition, growth, and mortality of Mojave desert tortoise (*Gopherus agassizii*) in the face of solar energy development. Biol. Conserv..

[65] Esque TC (2010). Effects of subsidized predators, resource variability, and human population density on desert tortoise populations in the Mojave Desert, USA. Endanger. Species Res..

[66] Lovich JE (2011). Effects of wind energy production on growth, demography, and survivorship of a desert tortoise (*Gopherus agassizii*) population in Southern California with comparisons to natural populations. Herpetol. Conserv. Biol..

[67] Harju SM, Cambrin SM, Averill-Murray RC, Fields KJ, Allison LJ (2020). Using incidental mark-encounter data to improve survival estimation. Ecol. Evol..

[68] Edwards T, Schwalbe CR, Swann DE, Goldberg CS (2004). Implications of anthropogenic landscape change on inter-population movements of the desert tortoise (*Gopherus agassizii*). Conserv. Genet..

[69] Riedle JD, Averill-Murray RC, Lutz CL, Bolen DK (2008). Habitat use by desert tortoises on alluvial fans in the Sonoran Desert, south central Arizona. Copeia.

[70] Todd BD (2016). Habitat selection by juvenile Mojave desert tortoises. J. Wildl. Manage..

[71] Jennings WB, Berry KH (2015). Desert tortoises (*Gopherus agassizii*) are selective herbivores that track the flowering phenology of their preferred food plants. PLoS ONE.

[72] Nafus MG, Esque TC, Averill-Murray RC, Nussear KE, Swaisgood RR (2017). Habitat drives dispersal and survival of translocated juvenile desert tortoises. J. Appl. Ecol..

[73] Dutcher KE, Nussear KE, Heaton JS, Esque TC, Vandergast AG (2023). Move it or lose it: Predicted effects of culverts and population density on Mojave desert tortoise (*Gopherus agassizii*) connectivity. PLoS ONE.

[74] United States Fish and Wildlife Service (USFWS). Passages for Connectivity of Mojave Desert Tortoise Populations Across Fenced Roads. United States Department of Interior. https://www.fws.gov/sites/default/files/documents/Passage%20spacing%20recommendations.27Mar2014.pdf (2014)

